# Prevention of leg cramps by using compression stockings or magnesium supplements in the 50–84 age group: study protocol for a randomised controlled trial

**DOI:** 10.1186/s13063-021-05753-0

**Published:** 2021-11-29

**Authors:** Jenni Joensuu, Pertti P. Mustajoki, Pekka K. Mustonen, Minna Kaila, Tuomas Koskela

**Affiliations:** 1 Tampere University, Tampere, Finland; 2 University Central Hospital of Helsinki, Helsinki, Finland; 3 The Finnish Medical Society Duodecim, Helsinki, Finland; 4 University of Helsinki, Helsinki, Finland

**Keywords:** Muscle cramp, Stockings, Compression, Magnesium

## Abstract

**Background:**

Leg cramps are painful sensations of tightening in the muscles of the legs that commonly appear during the night and are often associated with secondary insomnia. They are common especially in older age. There is no evidence that any method of prevention of nocturnal leg cramps is both safe and effective. There are no previous trials concerning cramp prevention by using compression stockings. The objective of this study is to assess in a prospective randomised controlled trial whether leg cramps can be prevented by the daily use of knee-length compression stockings or magnesium supplements.

**Methods:**

The study will be set in Finland, and 50–84-year-old volunteers will be recruited through Google Ads, the Finnish health library website and Finnish primary health care centres. The participants must have a minimum of two episodes of leg cramps per week for the past 4 weeks to be included in the study. The participants (*n* = 225) will be allocated to three equal groups: the compression stocking arm, the magnesium supplement arm and the placebo arm. The participants will go through 4 weeks of follow-up without intervention and then another 4 weeks of follow-up with the assigned intervention. The material for the study will be collected through electronic questionnaires.

**Discussion:**

This protocol describes a study that compares compression stockings, magnesium supplements and placebo for the prevention of leg cramps. The results of this study can significantly improve knowledge on the methods of preventing leg cramps.

**Trial registration:**

ClinicalTrials.gov NCT04694417. Registered on Jan 4, 2021.

## Administrative information

Note: the numbers in curly brackets in this protocol refer to SPIRIT checklist item numbers. The order of the items has been modified to group similar items (see http://www.equator-network.org/reporting-guidelines/spirit-2013-statement-defining-standard-protocol-items-for-clinical-trials/).

**Table:** 

Title {1}	Prevention of leg cramps by using compression stockings or magnesium supplements in the 50–84 age group: study protocol for a randomised controlled trial
Trial registration {2a}	ClinicalTrials.gov NCT04694417. Registered on Jan 4, 2021.
Protocol version {3}	12102021, version 2.0
Funding {4}	This study is funded by Lydia and Karl G. Lindberg’s Duodecim foundation.
Author details {5a}	Jenni Joensuu: Tampere University, Finland Pertti P. Mustajoki: University Central Hospital of Helsinki, Finland Pekka K. Mustonen: The Finnish Medical Society Duodecim, Finland Minna Kaila: University of Helsinki, Finland Tuomas Koskela: Tampere University, Finland
Name and contact information for the trial sponsor {5b}	Lydia and Karl G. Lindberg’s Duodecim foundation PL 713, 00101 HELSINKI, FINLAND
Role of sponsor {5c}	The funder of this study has no role in the study design, the writing of the report or the decision to submit the report for publication.

## Introduction

### Background and rationale {6a}

Leg cramps are sudden, painful episodes of involuntary tightening sensations that appear usually in the calf, thigh or foot muscles. The cramp lasts for an average of 9 min and can be relieved by powerful stretching of the affected muscle [1]. The symptom appears commonly during the night [1–3] and is often associated with secondary insomnia [1]. Some 30–50% of elderly outpatient clinic patients suffer from leg cramps, and less than half of them seek medical help because of these cramps [2, 3]. The incidence increases with age [3].

The exact aetiology is unclear. High-intensity exercises, nerve dysfunction and metabolic causes have been associated with nocturnal leg cramps [1]. Several medications and medical conditions have also been associated with muscle cramps [4]. Most cases are idiopathic [1].

Safe and effective treatments for nocturnal leg cramps have not been found [1]. Passive stretching, massage and mild exercises before going to bed are often used, but the evidence for their effectiveness is limited [1, 5]. Quinine has a modest effect on nocturnal leg cramps, but it is no longer in use because of drug interactions and serious adverse effects [6, 7]. The evidence for using magnesium to prevent nocturnal leg cramps has been contradictory [8, 9]. In a recently updated Cochrane systematic review, it was concluded that it is unlikely that magnesium supplementation provides cramp prophylaxis for older adults. However, there are no high-quality studies concerning exercise-associated muscle cramps or disease-state-associated muscle cramps [10]. Many other medications have also been suggested, such as carisoprodol, diltiazem, gabapentin, orphenadrine, verapamil and vitamin B_12_, but the evidence for their effectiveness is limited [1].

An article concerning cramps in the Finnish health library website for the general public [11] has yielded around 25 feedbacks from the readers, most of which highlight experience that using knee-length compression stockings could help to prevent nocturnal leg cramps. Compression stockings are not a standard of care currently, and there are no previous trials investigating the effectiveness of compression stockings for leg cramp prevention. There are no evidence-based guidelines on the treatment of leg cramps, but magnesium supplements are commonly used for this purpose. The main aim of this study is to assess the efficacy of knee-length compression stockings compared to magnesium supplements in a randomised controlled trial.

### Objectives {7}

The main outcomes are as follows:

1. The frequency of manifestation of the cramps.
2. The intensity of the cramps.
3. The quantity of nocturnal awakening because of the cramps.

### Trial design {8}

The study will be carried out as a randomised controlled trial. The participants will be randomised into three parallel treatment arms: compression stocking group, magnesium supplement group and placebo group. The groups will be of equal size. The participants will be blinded in the tablet arms. The framework is a superiority study between the three arms.

The participants will be recruited online via advertisements on Google and the national health library’s article on cramps. The outcomes of the study will be obtained using consecutive electronic questionnaires. The main outcome measures will be based on the participants’ record-keeping (collection of data on both symptoms and intervention adherence).

## Methods: participants, interventions and outcomes

### Study setting {9}

The study will be set in Finland and include voluntary participants, who will be recruited and followed online.

### Eligibility criteria {10}

Baseline information will be collected with an electronic questionnaire e-mailed to self-identified possible study subjects who have filled out an electronic contact information form. Based on the questionnaire, the researchers will decide whether the participants are suitable to participate in the study and the unsuitable individuals will be excluded at this stage. The subjects must have a functioning Internet connection and e-mail in active use to be able to participate.

#### Inclusion criteria

1. Minimum of 2 leg cramps per week in the past 4 weeks.
2. Age from 50 to 84 years.

A minimum of 2 leg cramps per week is thought to be sufficient for the response to treatment to be seen with this trial setting. Higher demand of symptoms could significantly complicate study recruitment. The age range of 50–84 is derived based on the perception that in younger people the aetiology of cramps is often different.

#### Exclusion criteria

- Peripheral artery disease (confirmed or suspected).
- Peripheral artery bypass surgery.
- Grave peripheral neuropathy or any sensory disorder.
- Allergy to the material of the compression stockings.
- Grave renal failure (GFR under 30 ml/min).
- The use of a magnesium carbonate product (e.g. Rennie® or Berocca®).
- Cardiac failure with pulmonary oedema or massive lower limb swelling.
- Lower limb soft tissue problems, including skin transplant, thinned skin, varicose ulcer, necrosis and any infection.
- Lower limb deformity or atypical shape or size that could prevent the usage of compression stockings.
- Continuous usage of compression stockings for any other reason than leg cramps.

### Who will take informed consent? {26a}

Participants included based on the criteria mentioned above will receive an e-mail, which contains information about the study protocol and the date when they are supposed to start observing their leg cramps daily. A consent form will be sent by post, along with a request to sign and return it to the principal investigator. Excluded individuals will also be informed by e-mail.

### Additional consent provisions for the collection and use of participant data and biological specimens {26b}

Not applicable.

### Pilot study

A pilot study was carried out from March 23 to August 9, 2020, with 12 participants. It showed that recruitment was slower than expected, this being the most important challenge of the protocol. Communication with the participants was fluent and engagement in the trial was very good—only one of the participants dropped out of the study. No significant side effects were observed. Minor amendments were made in the questionnaires after the pilot study based on feedback from the participants. Of the 12 participants, 11 would recommend participating in the study to a friend.

## Interventions

### Explanation for the choice of comparators {6b}

The comparators were chosen based on the popularity of magnesium supplements that are extensively marketed for cramp prevention, patient-reported experiences of compression stockings described above, and previous evidence (or lack of evidence). The placebo arm will act as a control group for the magnesium arm.

### Intervention description {11a}

#### Compression stockings

Compression stockings are mainly used for the prevention and reduction of lower limb oedema or venous thrombosis. There are three compression classes used in health care [12]. The compression stocking arm of the study will receive CE-marked stockings within the compression class 1 (20 mmHg or less compression). The correct size for the compression stockings will be defined by the reported circumference of the participant’s ankle and calf. If a participant measures the size incorrectly, a new pair of compression stockings will be sent to them in a different size based on their feedback. The participants will be given instructions to put the stockings on immediately after getting out of bed in the morning and to take them off before going to bed in the evening for the last 4 weeks of the study. Stockings within the mild compression class have no harmful effects on individuals when the exclusion criteria are considered and the size is configured appropriately [12]. The participants will be instructed to communicate with a dedicated research assistant via e-mail or phone in case of any problems or questions.

#### Magnesium

Magnesium is a mineral substance which regulates many biochemical reactions in the body, for example protein synthesis and the function of the muscles and nerves. It has a significant role in controlling blood sugar, blood pressure, energy generation and the formation of the bones. The recommended dietary allowance for magnesium is 420 mg for males and 320 mg for females over 50 years old. Dark green vegetables, leguminous plants, nuts, seeds and whole grains are good sources of magnesium [13, 14].

In the average Finnish diet, the recommendation is usually exceeded, and excessive amounts of magnesium in the body are extremely rare [15]. The magnesium arm of the study will take oral tablets containing 630 mg of magnesium hydroxide daily for the last 4 weeks of the study, which is equivalent to 250 mg of pure magnesium per day. The magnesium tablets for this study were manufactured and analysed by the Pharmia pharmaceutical company in Finland.

Magnesium does not accumulate excessively in the body through diet or oral supplements except in situations of renal failure, metabolic acidosis or advanced old age. Magnesium hydroxide tablets should not have any harmful effects on individuals when the excluding criteria are considered. The most common side effect of oral magnesium hydroxide is osmotic diarrhoea (1–2% of patients) or other irritation of the gastrointestinal tract [13, 14].

#### Placebo

The placebo tablets will consist of microcrystalline cellulose, magnesium stearate (anti-caking agent) and silicon dioxide. The placebo tablets were manufactured and analysed by the Pharmia pharmaceutical company in Finland. The placebo arm will receive placebo tablets to be taken daily for the last 4 weeks of the study. The participants will not know whether they are randomised into the magnesium arm or the placebo arm. The packaging and the appearance of the placebo and magnesium tablets are identical.

### Criteria for discontinuing or modifying allocated interventions {11b}

The allocated intervention can be discontinued at any stage of the study in response to harm or the participant’s request. Possible harms are, for example, an allergy to the material of compression stockings, allergy to an ingredient of either magnesium or placebo tablets, magnesium-induced diarrhoea or inability to find suitable compression stockings. Participants can drop out of the study at any time for any reason without consequences. Any harms reported to the research assistant will be recorded. Allocated interventions cannot be modified.

### Strategies to improve adherence to interventions {11c}

Our strategy to improve adherence during the 8 weeks of follow-up is to keep in active contact with the participants via a dedicated research assistant. The participants will receive an e-mail from the research assistant including a link to the weekly questionnaire once a week during the study. In case the questionnaire is not returned, the research assistant will contact the participant by e-mail and further by phone, when necessary.

### Relevant concomitant care permitted or prohibited during the trial {11d}

The use of any magnesium products or compression stockings other than those used as the interventions of the study is prohibited during the trial. Any other medications and supplements in use will be recorded and should be continued regardless of the study.

### Provisions for post-trial care {30}

Tampere University has continuous patient indemnity insurance that covers all its medical trials. If a participant experiences any harm related to this study, the indemnity is claimed from that insurance. It covers health care treatment injuries specified in the Finnish patient injury law. The Finnish Patient Insurance Centre is responsible for handling claims.

### Outcomes {12}

#### Primary outcome

1. The change in the quantity of leg cramps from the 4-week follow-up period (prior to intervention) to the 4-week intervention period.

The change in the quantity of leg cramps will be assessed based on the participants’ responses to the electronic questionnaires. The quantity of cramps will be recorded using a weekly online survey during the 4 weeks before intervention and during the 4-week intervention period. The difference between the number of cramps from the 4-week period prior to the intervention to the intervention period will be calculated.

#### Secondary outcomes

1. The change in intensity of leg cramps from the 4-week follow-up period (prior to intervention) to the 4-week intervention period.

The possible change in the intensity of leg cramps on a visual analogue scale from 1 to 10 will be assessed based on the participants’ responses to the electronic questionnaires. The intensity of cramps will be recorded using a weekly online survey during the 4 weeks before intervention and during the 4-week intervention period. The possible change in the intensity of cramps from the 4-week period prior to the intervention to the intervention period will be assessed.

2. The change in the quantity of nocturnal awakening due to the cramps from the 4-week follow-up period (prior to intervention) to the 4-week intervention period

The change in the quantity of nocturnal awakenings due to the cramps will be assessed based on the participants’ responses to the electronic questionnaires. The participants will record this data at the end of each week and are also instructed to keep their own cramp diary. The quantity of nocturnal awakening due to the cramps will be recorded using a weekly online survey during the 4 weeks before intervention and during the 4-week intervention period. The change in the number of nocturnal awakenings due to the cramps from the 4-week period prior to the intervention to the intervention period will be calculated.

### Participant timeline {13}

### Sample size {14}

The projected number of participants is based on sample size calculations, in which a power of 80% and significance level of 0.05 will show a difference between the intervention groups in the reduction of the quantity of leg cramps when variance analysis is used in statistical testing. The calculations for the minimum sample size are based on three of Cohen’s (1988) suggested effect sizes for *f* variance analysis. When the effect size of 0.25 and a drop-out rate of 30% are used, the number of applicable participants should be a minimum of 225 persons.

### Recruitment {15}

The participants will be recruited to the study by using Google advertisements and the Finnish health library’s website article on cramps [11]. The participants will also be recruited from Finnish primary health care centres. Additional information will be sent via e-mail to those interested and providing contact information. The additional information will include information on the study (including study protocol, possible advantages and downsides, informed consent) and a link to an electronic baseline questionnaire. A consent form will be sent by post along with a request to sign and return it to the principal investigator.

## Assignment of interventions: allocation

### Sequence generation {16a}

After completing the baseline evaluation, eligible participants will be allocated to the compression stocking group, the magnesium group or the placebo group in a 1:1:1 ratio by an independent investigator using a computerised random number generator. The allocation will be stratified concerning age. To prevent selection bias, the research assistant who takes care of posting the interventions will be the only person to know each participant’s group assignment prior to treatment. The randomisation has strict limits regarding authority; no one can check the files except the principal investigator.

### Concealment mechanism {16b}

The allocation will be made by an independent investigator who provides the random sequence and group assignment to the research assistant, who will be the only person to have the information. The recruiters assessing a study subject’s eligibility based on the baseline questionnaire will not be aware of the study group to which the next participant will be assigned to, so the recruiter’s decision to enrol a participant is not influenced by any knowledge of the group. The participants are unaware of the group in which they will be allocated when they join the trial, so the decision to provide informed consent is not influenced by the knowledge of the group to which they will be allocated. During the follow-up period prior to the intervention, the intervention allocation of the study participants will be informed to the research assistant who takes care of posting the intervention.

### Implementation {16c}

Participants will be enrolled by the research assistant and research teams who will assess a study subject’s eligibility based on the baseline questionnaire. The independent investigator will generate a randomisation sequence with a computerised random number generator and the block size of 3. The randomisation sequence and group assignment are then provided to the research assistant.

## Assignment of interventions: blinding

### Who will be blinded {17a}

The participants assigned to the two tablet groups will be blinded to the intervention. The magnesium and placebo tablets that are used in the trial will be identical in terms of packaging and appearance. The compression stocking group cannot be blinded, but the participants will not know their allotted intervention until they have received it by mail delivery. The statisticians will be blinded to the group allocation by using codes to label the three groups and will randomly assign outcome assessors to the follow-up. The allocation sequence will be concealed until the end of the study. The allocation code will be stored in a separate file.

### Procedure for unblinding if needed {17b}

Each participant will have a personal identification code that is asked in every questionnaire of the study. The identification code acts as a link between the study material and the participants’ identifying information. The independent investigator who has done the randomisation will know the allocated intervention and can unblind it if needed in case of serious adverse effects.

## Data collection and management

### Plans for assessment and collection of outcomes {18a}

**Fig. 1:**
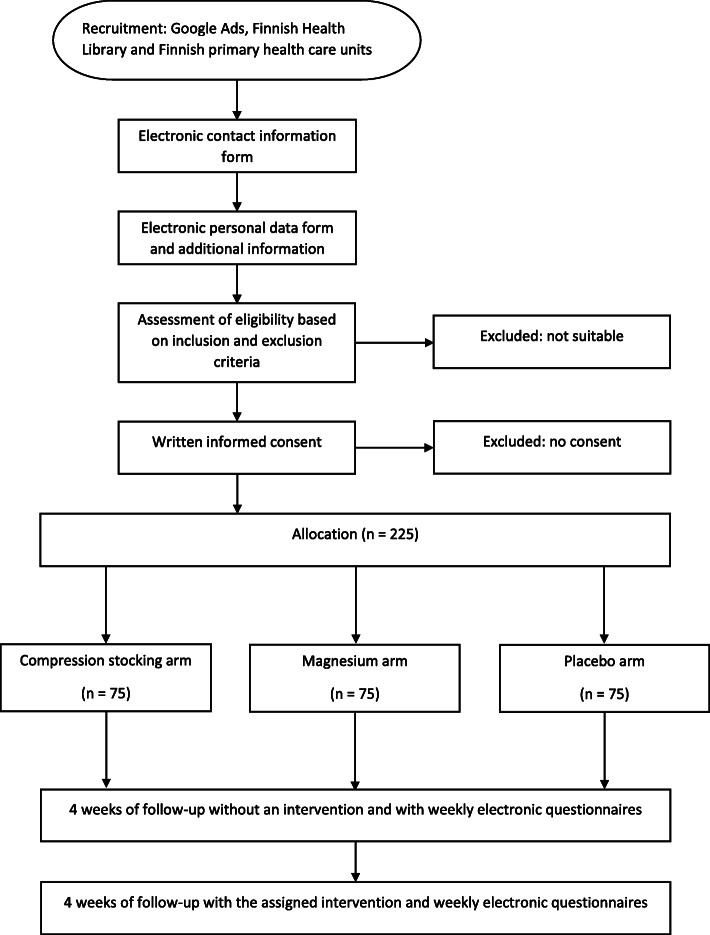
Participant timeline

**Fig. 2:**
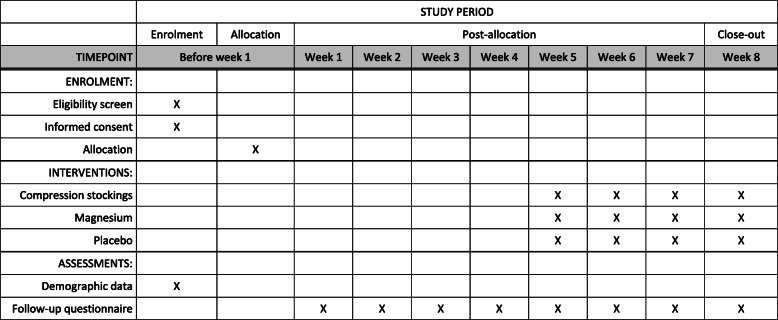
Schedule of enrolment, interventions, and assessments

The study will last for 8 weeks (Figs. 1 and 2). During the first 4 weeks, the participants will observe the frequency of the manifestation of the cramps, the intensity of the cramps and the quantity of nocturnal awakening due to the cramps without any treatment. Information will be collected at the end of each week using an electronic questionnaire. A link to the questionnaire will be sent weekly by e-mail as a reminder. Participants will be requested to keep a tally of the leg cramps daily in order to improve the reliability of the results by minimising recall bias. The compression stockings, magnesium tablets and placebo tablets will be posted to the participants along with instructions for use. During the following 4 weeks, each of the participants will observe the incidence of the cramps, the intensity of the cramps and the quantity of nocturnal awakenings due to the cramps using the intervention that they have been sent in accordance with randomisation. The weekly reporting of the symptoms will continue using the same electronic questionnaire as previously. Any other symptoms, participant’s health or changes in the way of life will not be monitored during the follow-up.

### Plans to promote participant retention and complete follow-up {18b}

Demographic data from all the participants will be collected through a baseline questionnaire. Weekly questionnaires are collected until possible discontinuation. In order to prevent dropouts, the research assistant will remind participants weekly by e-mail to fill out the questionnaire and will contact them if a weekly questionnaire is not returned. The participants will be instructed to communicate with the research assistant via e-mail or phone in case of any problems or questions related to the study.

### Data management {19}

All data will be collected electronically without any interphases by the participants through Microsoft Forms® electronic forms. Any Excel files will be formed without manual data recording.

### Confidentiality {27}

Material for the study will be collected using electronic questionnaires. Participants will fill out the questionnaire themselves once every week for 8 weeks. The consent forms that contain the participants’ names, birth dates or social security numbers, addresses, phone numbers and e-mail addresses will be sent to the principal investigator and stored in a locked cabinet of a locked office at the Unit of General Practice at Tampere University. The study material will be stored separately on secured computers, and it will not contain any information that could reveal the identity of the participants. In addition, the contact information form will be stored separately on the computers. Only the principal investigator and the research assistant will have access to the documents. Each participant will have a personal identification code that can be used to connect the study material to the identifying data. The identifying data, consent forms and study material will be destroyed 10 years after the beginning of the study.

### Plans for collection, laboratory evaluation and storage of biological specimens for genetic or molecular analysis in this trial/future use {33}

Not applicable, no samples collected.

## Statistical methods

### Statistical methods for primary and secondary outcomes {20a}

Descriptive statistics of the demographics will be displayed to ascertain any marked imbalance between the trial arms at baseline. The results of each of the three intervention groups will be analysed separately and compared. The primary comparative analysis will be conducted on an intention-to-treat-analysis.

The primary outcome will be the difference in the quantity of the leg cramps between the first and the second 4-week periods of the study comparing the three trial arms, and it will be analysed by one-way analysis of variance.

The secondary outcomes will be (1) the change in the intensity of the cramps between the first and the second 4-week periods of the study comparing the three trial arms and (2) the awakenings due to cramps between the first and the second 4-week periods of the study comparing the three trial arms. The difference between groups is analysed by a one-way analysis of variance of repeated measurements.

### Interim analyses {21b}

There are no plans for interim analyses.

### Methods for additional analyses (e.g. subgroup analyses) {20b}

There are no plans for additional analyses.

### Methods in analysis to handle protocol non-adherence and any statistical methods to handle missing data {20c}

Feedback from participants and reasons for dropping out will be analysed. Sensitivity analysis using multiple imputation models for missing values will be conducted to investigate the potential effects of the missing data.

### Plans to give access to the full protocol, participant-level data and statistical code {31c}

The full protocol will be available in open access format. The datasets generated and analysed during this trial will be available from the corresponding author on reasonable request.

## Oversight and monitoring

### Composition of the coordinating centre and trial steering committee {5d}

The research group will act as a trial steering committee.

### Composition of the data monitoring committee, its role and reporting structure {21a}

An external steering group was deemed impracticable and unnecessary, as this is a one-site trial on previously (for a variety of purposes) used interventions.

### Adverse event reporting and harms {22}

Based on the literature and previous experience, only minor adverse events are expected. Any adverse events and harms reported to the research assistant will be discussed immediately within the research group.

### Frequency and plans for auditing trial conduct {23}

This trial is independent of its sponsors. The research group complies with the protocol and the usual ethics practices. There are no planned procedures for auditing trial conduct.

### Plans for communicating important protocol amendments to relevant parties (e.g. trial participants, ethical committees) {25}

Any major protocol modifications will be discussed within the research group and with the local ethics committee according to available national and local guidelines. The participants will be informed of the modifications.

### Dissemination plans {31a}

The results of this study will be reported internationally in appropriate journal series, conference presentations and other forms, whether they are positive or negative. The participants will receive a summary of the results if they requested a summary in the contact information form.

## Discussion

This protocol describes a study that compares compression stockings, magnesium supplements and placebo for the prevention of leg cramps. Leg cramps are a common symptom that is often associated with secondary insomnia [1–3]. Magnesium supplements are commonly used for this purpose even though it is unlikely to benefit from them [10]. According to PubMed, there are no studies concerning compression stockings for the prevention of leg cramps whatsoever despite the patient-reported experience. The results of this study can significantly improve knowledge on the methods of preventing leg cramps.

### Strengths

The strengths of this study are that the participation does not require any appointments with health care or the researchers and the interventions have no serious effects on individuals in general when compliance with the exclusion criteria is ensured. Communication with the research assistant is made easy with a direct e-mail address and phone number, which should be used at a low threshold. Additionally, gathering evidence and experience on the conducting of the study using data on Patient-Reported Outcome Measures (PROM) and Patient-Reported Experience Measures (PREM) as recorded by the participants themselves is significant. Results from the undertaken pilot study support the feasibility and acceptability of this protocol.

### Limitations

The limitations of this study are the inability to blind the compression stocking arm during the intervention stage and the relative length of the study, as the follow-up lasts for 8 weeks. Putting on a compression stocking can also seem to be challenging at first and affect the engagement with the study. The participants must have a functioning internet connection and e-mail address in active use to be able to participate and that can limit especially older people’s opportunities to participate and increase selection bias. Recruitment via online advertisement can also lead to bias. A person searching for information about leg cramps on the Internet can be more health-conscious, have more pronounced symptoms or be less likely to have other interventions affecting the symptoms. The reliability of the material is based on the participants’ own evaluation and record-keeping of the symptoms and usage during the intervention, so the data are not objective. If a participant does not follow the directions of keeping a tally of the symptoms daily and reporting them weekly via the electronic questionnaires, there is an increased risk of recall bias.

### Trial status

At the time of submission, a pilot study (12 participants) has been completed. The participant enrolment for the trial has started in May 2021.
